# Freiburg Neuropathology Case Conference: Headache, Mental Confusion and Mild Hemiparesis in a 68-year-old Patient

**DOI:** 10.1007/s00062-023-01359-y

**Published:** 2023-10-23

**Authors:** M. Frosch, T. Demerath, C. Fung, M. Prinz, H. Urbach, D. Erny, C. A. Taschner

**Affiliations:** 1 Department of Neuropathology, University of Freiburg, Freiburg, Germany; 2 Department of Neuroradiology, University of Freiburg, Freiburg, Germany; 3 Department of Neurosurgery, University of Freiburg, Freiburg, Germany; 4 Medical Centre—University of Freiburg, Faculty of Medicine, University of Freiburg, Breisacherstr. 64, 79106 Freiburg, Germany

**Keywords:** Glioblastoma, Gliosarcoma, Cerebral metastasis, Primary CNS lymphoma, Pleomorphic xanthoastrocytoma

## Case Report

A 68-year-old female was admitted to the emergency department due to headache, mental confusion and a mild hemiparesis on the left side in October 2021. Her medical history revealed dilatative cardiomyopathy, arterial hypertension and nicotine abuse but no known malignancy. Clinical evaluation yielded a hemiparesis on the left side grade 4 according to the Medical Research Council (MRC) scale. Magnetic resonance imaging (MRI) was performed and showed a right temporal mass lesion with perifocal edema. The case was discussed at the interdisciplinary neuro-oncological board and surgery was indicated.

At surgery a temporal craniotomy was performed followed by a temporal pole resection with resection of the temporomesial structures. The surgery was uneventful and the patient had no new neurological deficits. A postoperative MRI scan showed a complete resection of the contrast-enhancing portions. The patient was transferred to an oncology ward. Due to renal insufficiency, the chemotherapy could only be started with a reduced dose of cytarabine and without methotrexate. The chemotherapy was well tolerated. No tumour recurrence has been detected in the regular follow-up MRIs to date.

## Imaging

**Fig. 1:**
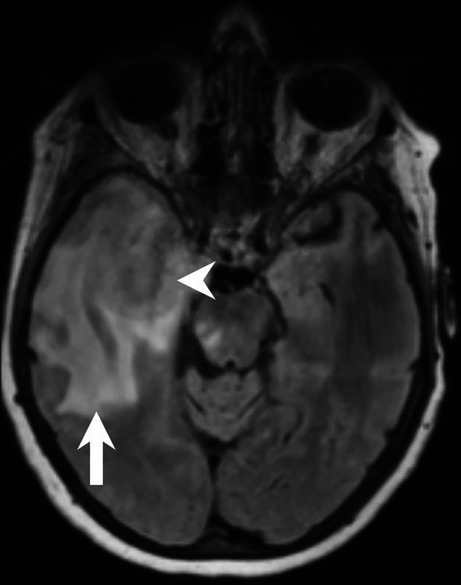
Axial fluid-attenuated inversion recovery (FLAIR) image of the head showed a large space-occupying intra-axial lesion of the right temporal lobe. The lesion (*arrowhead*) could hardly be discriminated from the extended perifocal edema (*arrow*)

**Fig. 2:**
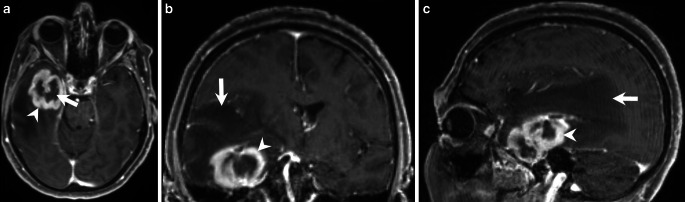
On axial (**a**), coronal (**b**), and sagittal (**c**) T1-weighted images taken after administration of gadolinium (Gd), the lesion showed distinct, giriform contrast enhancement (**a**–**c**, *arrowheads*) with signs of central necrosis (**a**, *arrow*). Please note the large perifocal edema (**b, c**, *arrow*)

**Fig. 3:**
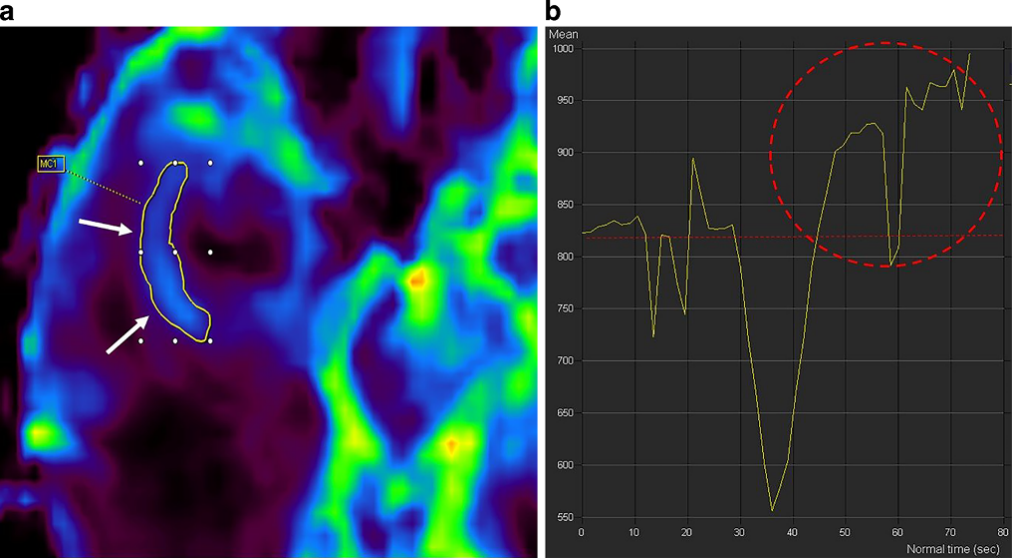
On dynamic susceptibility-weighted, contrast-enhanced (DSC) perfusion MRI the lesion showed increased perfusion on the cerebral blood volume (CBV) map in axial orientation (**a**, *arrows*). The signal intensity-time curve (**b**), derived from a region of interest within the CBV map, markedly exceeded the baseline after the first pass (**b**, *red circle*), a finding considered characteristic for primary cerebral lymphomas [14]

The MRI showed a large space-occupying intra-axial lesion of the right temporal lobe (Figs. 1, 2 and 3). On the fluid-attenuated inversion recovery (FLAIR) images the lesion (Fig. 1, arrowhead) could hardly be discriminated from the extended perifocal edema (Fig. 1, arrow). On T1-weighted images after administration of gadolinium (Gd), the lesion showed distinct, giriform contrast enhancement (Fig. 2a–c, arrowheads) with signs of central necrosis (Fig. 2a, arrow). Note the large perifocal edema (Fig. 2b, c, arrow). On diffusion-weighted MRI (b-value: 1000) the lesion did not display any signs of restricted diffusion (not shown). On dynamic susceptibility-weighted, contrast-enhanced (DSC) perfusion MRI the lesion showed increased perfusion on the cerebral blood volume (CBV) map (Fig. 3a, arrows). The signal intensity-time curve (Fig. 3b), derived from a region of interest (ROI) within the CBV map, markedly exceeded the baseline after the first pass (Fig. 3b, red circle).

## Differential Diagnosis

### Glioblastoma

Glioblastomas (GBM, IDH wildtype) are the most common primary central nervous system (CNS) tumors in adults, mostly arising supratentorially with a preponderance in men in the 7th decade of life [1]. According to the recent World Health Organization (WHO) criteria they represent high-grade, rapidly growing, diffusely infiltrating astrocytic tumors, classified as CNS WHO grade 4 neoplasms, with a poor prognosis [2, 3]. Due to aggressive and rapid growth, glioblastomas usually show signs of neovascularization and necrosis, surrounded by diffuse tumor infiltration [4]. Typical findings in MRI are irregular “shaggy” enhancing margins with a central necrotic core, surrounded by large T2/fluid-attenuated inversion recovery (FLAIR) hyperintense signal alteration and diffuse non-enhancing tumor infiltration [4]. Given the large temporal intra-axial mass with irregular rim enhancement and the incidence of these tumors in adults between the 6th and 7th decades of life, glioblastoma was considered as the favorite differential diagnosis in this case.

### Gliosarcoma

Gliosarcoma (WHO grade 4) is defined as a subtype of IDH wildtype GBM, histologically characterized by a biphasic tissue pattern with glial and mesenchymal differentiation [2, 5]. In MRI, gliosarcomas may either present with a predominant sarcomatous components as a homogeneous Gd-enhancing mass, or with a predominant gliomatous component, resembling GBM [2, 6]. Extracranial involvement in gliosarcomas has been reported in the minority of cases [6]. The superficial location in this case with potential meningeal and ependymal involvement makes gliosarcoma a relevant differential diagnosis in this case.

### Metastasis

Metastases represent the most common secondary CNS tumor in adults with roughly 1/3 of all intracranial neoplasms, commonly arising from lung or breast carcinoma or malignant melanoma [7]. They may present as solitary lesion in up to half of cases [7], typically occurring at the gray-white matter junction and may also present with intratumoral hemorrhage [8]. Surrounding edema is often disproportional to the central tumor component [8]. The incidence of cerebral metastases in middle-aged adults and their heterogeneous imaging pattern makes them a relevant differential diagnosis in patients with solitary intra-axial, Gd-enhancing mass lesions. This is also why brain metastasis is certainly a relevant differential diagnosis in this case.

### Primary CNS Lymphoma

Primary central nervous system lymphomas (PCNSL) are relatively rare, accounting for only 4% of all intracranial neoplasms, with a peak incidence at about 60 years [2, 9, 10]. Cerebral lymphomas mostly represent mature B‑cell lymphomas [2] and typically present as a hypercellular mass with vivid and homogeneous enhancement after contrast administration, often with restricted diffusion on diffusion weighted imaging (DWI) [11] but may also present with central necrosis, which has been related to Epstein-Barr virus (EBV) positivity [12]. While a solitary manifestation can be observed in the majority of cases, PCNSL may present with multifocal manifestations in about 30–40% of patients [11], often involving the periventricular white matter [2], which is uncommon for brain metastases or GBM. The differentiation between GBM and atypical PCNSL can be improved by using dynamic susceptibility-weighted, contrast-enhanced (DSC) perfusion MRI or even multiparametric approaches [13–15]. As this lesion presented as solitary lesion with a large necrotic core within the temporal pole, PCNSL was not considered as the primary differential.

### Pleomorphic Xanthoastrocytoma

Pleomorphic xanthoastrocytomas (PXA) WHO grade 2–3 are rare astrocytic tumors accounting for 1% of primary brain tumors, usually seen in the pediatric and young adult population. PXAs typically occur in the supratentorial brain with a predilection for the temporal lobe, commonly related to temporal lobe epilepsy. With a mostly superficial localization, a cyst with Gd-affinitive nodules is often part of the lesion, which in addition often presents with an adjacent lepromeningeal and/or dural Gd-enhancement [2, 16]. Due to long-standing superficial growth, skull remodeling can be found [2, 16]. Regarding the described imaging features of PXA, the diagnosis seems less likely in this older patient with a primary central necrotic lesion, without a circumscribed nodular or cystic tumor component; however, due to the superficial location in the temporal lobe, the diagnosis of PXA needs to be considered.

## Histology and Immunohistochemistry

**Fig. 4:**
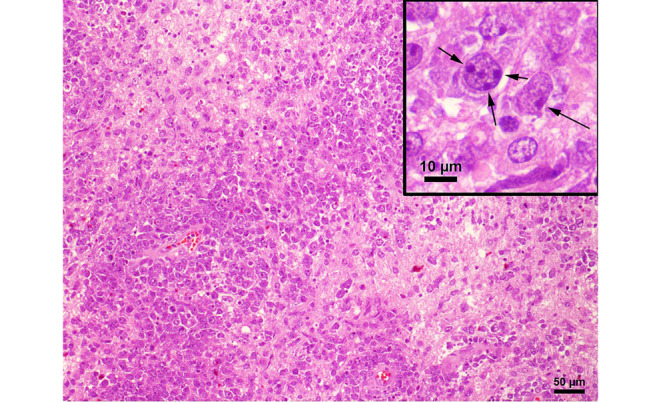
Hematoxylin and eosin (H&E) stain that showed a highly cellular, diffuse growing tumor. The neoplastic cells exhibited a relatively large, round to oval, irregularly shaped nucleus. Some cells showed a cytoplasm of variable size. The higher magnification (insert) depicted tumor cells with multiple nucleoli stuck to the nuclear membrane (*arrows*). Scale bar: 50 µm and 10 µm

**Fig. 5:**
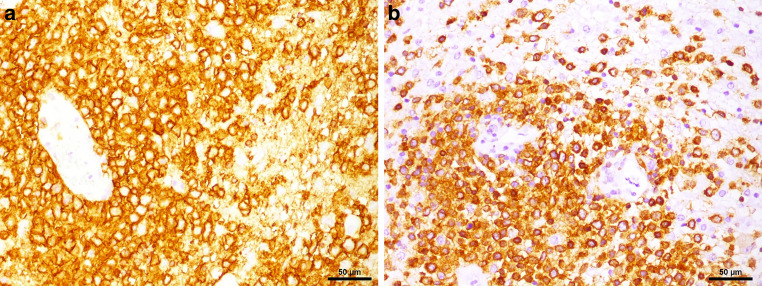
CD20 immunoreactivity (**a**) was observed in all tumor cells. Moreover, most tumor cells expressed CD79a (**b**). Scale bar: 50 µm

**Fig. 6:**
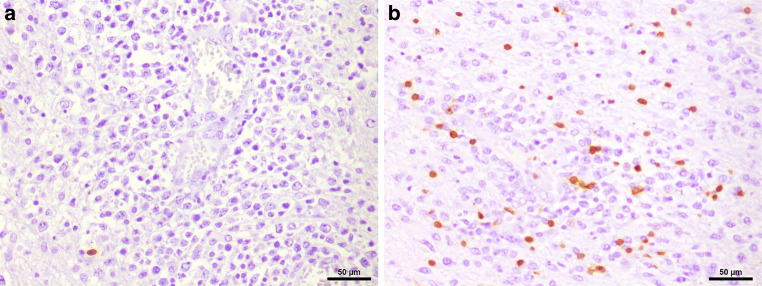
CD38 (**a**) and CD3 (**b**) were only present in a few scattered non-tumor cells. Scale bar: 50 µm

**Fig. 7:**
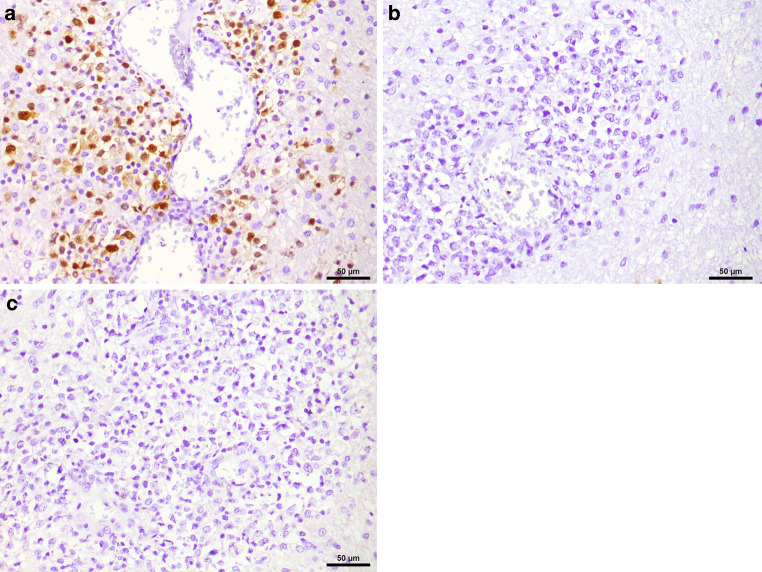
The tumor cells exhibited expression of MUM1 (**a**) but not CD10 (**b**) and BCL6 (**c**), indicating a non-germinal center B‑cell (GCB) lymphoma according to the Hans algorithm, Scale bar: 50 µm

**Fig. 8:**
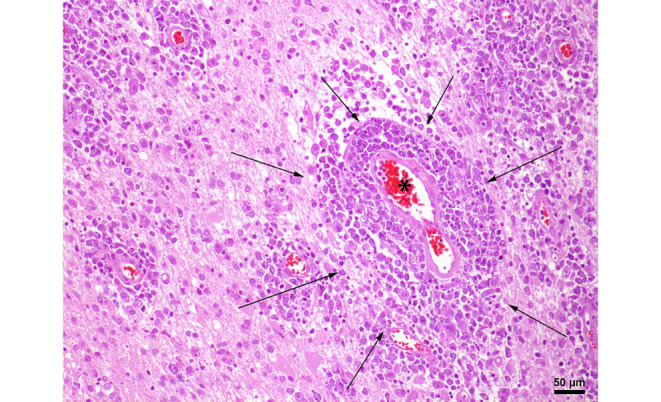
Hematoxylin and eosin (H&E) stain that showed tumor cells growing in an impressive angiocentric pattern (*arrows*). The *asterisk* highlights the vessel lumen; *arrows* point toward the border between tumor cells and necrotic tissue. Scale bar: 50 µm

**Fig. 9:**
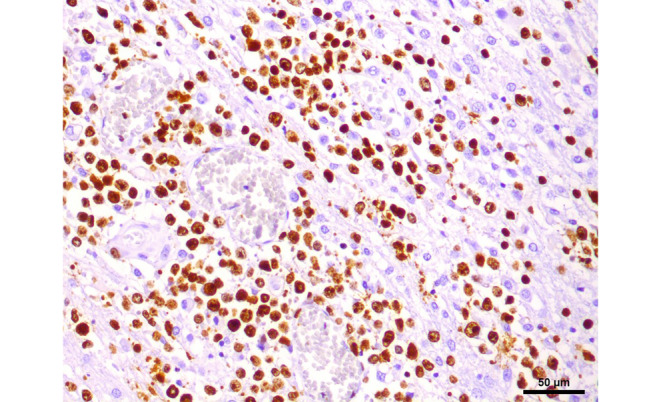
Staining for the proliferation marker Ki-67 showed more than 80% positive tumor cells. Scale bar: 50 µm

In the hematoxylin and eosin (H&E) stained sections of the formaldehyde-fixed and paraffin-embedded biopsy material, fragments of a highly cellular, diffusely growing tumor with many medium to large sized lymphoid cells were found (Fig. 4). These lymphoid cells exhibited a relatively large, round to oval nucleus with prominent nucleoli, often more than one sticking to the nuclear membrane (Fig. 4). In addition, some tumor cells showed a well-circumscribed cytoplasm of variable size, making them look lymphoblastic-like; however, the tumor cells were mature B cells, labelled positive in the immunohistochemical staining of CD20 and CD79a (Fig. 5). In contrast, T cell markers (CD3) or plasma cell markers (CD38) remained negative within the tumor cells and only stained single scattered cells (Fig. 6). Up to 60% of the tumor cells exhibited expression of MUM1, but expression of CD10 and BCL6 was absent (Fig. 7). BCL2 is expressed by around 80% of the tumor cells; CMYC overexpression is, however, not present. In addition, EBV antigens could not be detected immunohistochemically. Of note, the tumor cells grow in a clearly angiocentric pattern and lie in a dense and diffuse manner next to each other (Fig. 8). The vessels appeared to be split with lymphoid infiltrates. Isolated vessels are clogged. Between these perivascular tumor nodes, necrotic zones appear (Fig. 8). Moreover, larger necrotic areas are found at many sites next to vital tumor tissue. Apoptotic cell bodies can be found, and mitotic activity is brisk; many mitotic figures appear within the tumor. In line with high proliferative activity, up to 80% of the tumor cells are marked in the immunohistochemical staining against Ki-67 (Fig. 9). In summary, the histopathological finding of a highly cellular, highly proliferative, diffusely growing tumor with large B cells (CD20 + CD79+) primarily appearing within the temporal lobe leads to the final diagnosis.

In summary, the histopathological finding of a high cellular, highly proliferative, diffuse growing tumor with large B cells (CD20 + CD79+) primarily appearing within the temporal lobe leads to diagnosing a primary diffuse large B‑cell lymphoma of the CNS.

## Diagnosis

### Primary Diffuse Large B-cell Lymphoma of the CNS (CNS-DLBCL)

Diffuse large B‑cell lymphoma of the CNS (CNS-DLBCL) is a rare tumor that accounts for 1–3% of primary CNS tumors but is the most common tumor type encountered among primary CNS lymphomas [17, 18]. They typically occur in the cerebral hemispheres (38%) and are less frequent in the thalamus, basal ganglia or cerebellum [19]. For CNS-DLBCL, neither etiological nor genetic predispositions have been described [2]. Macroscopically, CNS-DLBCLs are quite firm and granular, and often exhibit central necrosis [2] as observed in the present case. In general, lymphomas are characterized by nutrient deprivation and hypoxia ultimately leading to tumor necrosis [20]. Interestingly, the presence of necrosis in CT or FDG-PET can serve as a prognostic marker. Patients with tumor necrosis have a significantly worse outcome than those without tumor necrosis [21, 22]. Histologically, tumor cells conform to mature B cells (late germinal center exit), and thus they express typical mature B cell markers such as CD20, CD19, PAX5, or CD79a. In the presented case CD10 expression of the DLBCL was negative arguing against a systemic DLBCL manifestation. CNS-DLBCLs have a significantly worse outcome than systemic DLBCL [23]. The mechanism behind the poor prognosis of CNS-DLBCL is not fully understood. CNS-DLBCLs appear to co-express BCL2, BCL6, and/or MYC at higher frequencies than systemic DLBCL [24]. This co-expression is an established prognostic risk factor [25, 26] and, thus, could point towards potential mechanisms underlying the adverse prognosis of CNS-DLCBL; however, in this case we only found overexpression of BCL2 suggesting a potentially better prognosis. Important differential diagnoses of the CNS-DLBCL include other CNS lymphomas, such as immunodeficiency-associated CNS lymphomas, intravascular large B‑cell lymphoma, or T‑cell and NK/T-cell lymphomas; however, approximately 95% of all primary CNS lymphomas are DLBCL. Only 5% include the mentioned differential diagnosis, and thus, they are exceptionally rare [27].
